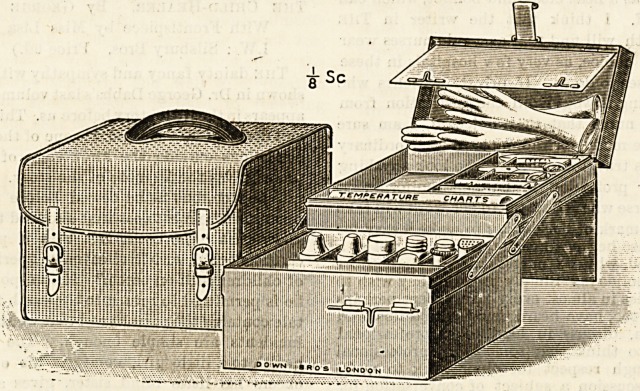# The Hospital. Nursing Section

**Published:** 1902-08-02

**Authors:** 


### The Hospital.

### Nursing Section.

**Contributions for this Section of "The Hospital" should be addressed to the Editor, "The Hospital" Nursing Section, 28 & 29 Southampton Street, Strand, London, W.C.**

No. 827.—Vol. XXXII. *SATURDAY, AUGUST* 2, 1902.

### Notes on News from the Nursing World.

### THE QUEEN AND THE IMPERIAL YEOMANRY NURSES.

The sisters on the staff of the Imperial Yeomanry Hospitals who distinguished themselves during the war will have the great pleasure of receiving their medals from the Queen, who has graciously consented to distribute them in the gardens of Devonshire House, Piccadilly, on Monday, August 11th, at 4 p.m. It will be recollected that there was a reception of the members of the staff at Devonshire House before they went out to South Africa, and it is in accordance with the fitness of things that the honours awarded for services faithfully rendered should be bestowed in the same place. Any nurses belonging to the staff, who have not had the official notice, should communicate with Mr. Oliver Williams, 116 Victoria Street, Westminster, S.W., when a card of admission to the ceremony will be forwarded.

### THE KING'S NURSES.

A ridiculous complaint is made that there has been incomprehensible reticence about the names of the nurses who have been attending the King during his illness. It has, on the contrary, appeared to us that unusual publicity has been given to the names of those ladies. For instance, the portrait and biography of a nurse who at the outset rendered some assistance has been published in a number of newspapers. The only nurses who have been in constant attendance upon his Majesty are, as we have already intimated, Miss Fletcher, a native of Lancashire, who was trained at the Hospital for Women, Shaw Street, Liverpool, and Miss Haines, a native of county Cork, who was trained at the London Hospital.

### THE WOMEN'S MEMORIAL TO QUEEN VICTORIA.

The respectable sum of £487 8s. 3d. has been collected in Monmouthshire towards the Women's Memorial to Queen Victoria. Lady Llangattock, by whom the amount was forwarded to the head office, has received the thanks of the committee for the contribution. It has been a feature in Monmouthshire, as in other counties, that the shillings and pence of the poor have been subscribed by people anxious at once to show their respect for the late Sovereign and to assist the movement for increasing the number of Queen's Nurses. We are glad to learn that the movement has been taken up with enthusiasm in Johannesburg, South Africa, and that there is every probability that the branch will make a considerable contribution to the Fund.

### MENTIONED IN LORD KITCHENER'S DESPATCH.

The final despatch of Lord Kitchener, which was published on Wednesday, contains the names of a large number of persons whose "kind assistance and loyal service" during his command in South Africa he desires to recognise. The list includes the following members of the Army Nursing Service :—Superintending Sisters J. A. Gray and M. Russell, Sisters M. C. S. Knox, C. H. Keer, H. L. Neale, E. J. Martin, G. E. Saunder, A. A. Murphy, A. C. L. Anderson, A. R. Rose-Innes, and A. E. Tait; of the Army Nursing Service Reserve :—Sisters H. O. Luckie, L. Mitchell, G. Black, A. F. Bedwell, J. Creighton, C. M. Jones, G. Roberts, E. M. Monck-Mason, and J. Halliday; and of nursing sisters in various Colonies :—Superintendent M. Rawson (Victorian), Sister Isobel Ivey (Victorian), Sister M. S. Bidsmead (Australian), Sister D. Peiper (New Zealand), and Sister A. V. Pocock (New South Wales).

### NURSES FROM SOUTH AFRICA.

The members of Queen Alexandra's Imperial Nursing Service who arrived at Southampton in the *Harlech Castle* on Thursday last week were Sisters M. S. Barwell and S. G. Willetts, who are not required to return to South Africa owing to the reduction of the establishment. On the *Avondale Castle* which arrived last Friday were Sisters N. S. Brown, and C. Thomas, who were sent home on duty, and are not required to return. Both the *Briton* and the *Gaika* arrived on Saturday. The former had on board Sisters C. E. E. Marsh and E. E. Baldrey; and the latter, Sisters A. Luscombe and M. Holloway, neither of whom return. The *Goorkha*, which left Capetown on July 19th and is due at Southampton August 10, has on board Sisters A. Pallot, F. E. Filkin, A. E. French, A. Murton, M. E. Powell, and S. C. Schlieman. The *Alnwick Castle*, which is due August 11, will have on board Sisters C. M. C. Rogers and E. Lawson; and *Fort Salisbury*, which is due August 16, Sisters F. N. Davies and A. G. Wylie.

### NURSING IN AN EGYPTIAN QUARANTINE STATION.

In view of the serious outbreak of cholera in Egypt, the article by a member of the nursing staff at the quarantine station of El Tor, which appears in another part of the paper, will be read with special interest. So far as the pilgrims to Mecca are concerned, it appears that every possible precaution was taken to prevent the introduction of the disease. The great heat at Tor seems to have been the most serious drawback under which the sisters laboured during their sojourn at the quarantine station, the number of patients being too great to allow sufficient opportunities for rest. Another difficulty to contend with was that some of the people not only declared that they wished to die, but did their best to make it impossible for them to recover. However, in spite of these, and other disadvantages, the experience to be gained in such circumstances may have rendered the work attractive.

### A SUCCESSFUL BOER NURSE.

General Lucas Meyer, who is now in England with his wife, is also accompanied by his daughter. To Miss Meyer belongs the honour of having rendered valuable services as a nurse in the early stages of the late war. Her capacity may be judged by the fact that, after the British occupation of Pretoria, she continued to nurse in the Boer hospital. One of our officers who rendered gallant service in the field and was badly wounded gives Miss Meyer credit for having saved his life by her ministrations. In view of some of the criticism which it has been necessary to pass upon Boer nursing, it is a pleasure to hear of Miss Meyer's emphatic success.

### THE TRAINING AND SUPPLY OF MIDWIVES.

The final meeting of the Association for Promoting the Compulsory Registration of Midwives was held last week. But though the Association, its work accomplished, has been dissolved, it has wisely been determined to form a new body who will concern themselves with organising the training and supply of midwives. In this direction there is still ample scope for the energies of Mr. Heywood Johnstone, M.P., and his friends. Unless the midwives of the future are adequately trained, the new Act of Parliament will not be of much practical benefit to the community.

### HOSPITAL NURSES' COSTUMES PROVIDED BY SOAPMAKERS.

It does not require to be proved that the wearing of the uniform of a hospital nurse is no proof of the possession of hospital training. But it has been reserved for a firm of soapmakers at Bootle to abuse the uniform in the most unblushing and outrageous manner. Advertising in a Manchester paper for "lady canvassers for Blackburn, to call from house to house," for the purpose of introducing their soap, they offer as inducements, "wages 21s. per week; hospital nurses' costumes provided by us." The law, of course, does not prevent this degradation of the uniform of hospital nurses, and we are afraid that a sufficient number of young women who wish to earn 21s. a week will jump at the opportunity of masquerading in a costume to which they have no professional title. But we certainly hope that the firm which exposes them to the temptation will find that the public refuse to purchase an article which is advertised by means that constitute an insult to the nursing profession throughout the country.

### HEYWOOD NURSING ASSOCIATION.

A feature of the annual meeting of the Heywood Sick Nursing Association was that thanks were voted to a great variety of bodies who had afforded help and co-operation during the twelvemonth, including the Corporation, the Bury Board of Guardians, the Heywood Co-operative Society, the Tramway Company, the promoters of a concert held last March, the wardens of several churches, and the Heywood Wheelers Cyclist Club. Thanks to this assistance, the balance at the bank was only slightly reduced in order to meet the expenditure. As to the work done by the nurses, upwards of 10,000 visits were paid, and 284 cases were dealt with—a record which will doubtless tend to increase the local support given to the Association.

### NURSING SMALL-POX PATIENTS ON THE HAMPSHIRE DOWNS.

After the numerous letters which we have received from time to time dilating on the hardships and horrors of private nursing, the following account, written by one of two nurses who are undergoing a novel experience and accepting all their discomforts in a happy and cheerful manner, is distinctly refreshing. "We are having such exciting times, living in two tents—one for a bedroom, the other for a ward—and a little shed for our kitchen. A man lives in a tent at the bottom of the field, and does our shopping for us. Of course we do all our own cooking, etc., but we have a very nice stove. The first night we were here for supper, we could not find any knives, forks, or spoons. Luckily, one of us had a knife and fork, so we managed with that, and after breakfast next morning we found the others in the slop pail! The kitchen is built of wood, and contains a cooking stove, cupboard, table, and two chairs, and all the cooking utensils are quite new; so you see we are quite comfortable. We have an immense covered tank of water outside, and the man brings us up fresh drinking water every day. We are always killing earwigs and still there seem more to kill. Our patient has small-pox very badly, and we expect more patients every day."

### COMPLIMENT TO A MATRON AT EXMOUTH.

The annual report of the Hume-Long Hospital at Exmouth is of a gratifying character. Last year 75 in-patients and 104 out-patients received treatment, and the committee start a new year with a credit balance of £84. This, it is stated, is largely due to efficient management and the curtailment of the expenses of administration since the appointment as matron of Miss Jeffery, who was previously at Cheltenham Infirmary and was also at Newton Abbot Workhouse Infirmary for several years as superintendent nurse. The committee affirm that the highest praise is due to her for the care and attention the patients have received. Before another annual meeting comes round it is hoped that the new building, which is to be known as the Exmouth Cottage Hospital, will have been completed.

### A CONSIDERATE BOARD OF GUARDIANS.

It is always pleasant to record an act of kindness and consideration on the part of a Board of Guardians. Miss Ringrose, who has been for the last three and a half years superintendent at Christchurch Workhouse Infirmary, has, unfortunately, had a very serious illness. It being the opinion of the medical officer that a complete rest was necessary for her restoration to health, the Christchurch Board of Guardians have granted her six months' leave of absence with salary during that period. We are not surprised to hear that the desire of the Board to do their utmost for the welfare of their nurses, expressed by this act of practical sympathy shown to the chief, is much appreciated by the whole of the staff.

### WEST OF ENGLAND NURSES IN CONCLAVE.

In accordance with a suggestion made at an entertainment recently given to nurses by Mrs. Martin Gibbs, that district, workhouse infirmary, and private nurses in the West of England might find it helpful to them in their work to meet together from time to time for the purpose of discussion, the first of these gatherings took place on Thursday last week in the Wych Room, Long Ashton. The attendance was small, though a number of invitations were sent out; but there was an interesting discussion on "maternity work in connection with district nursing, and the desirability of having maternity cottage hospitals for country districts." It is hoped that the next meeting may be arranged for early in October, at Weston-super-Mare.

### THE TRAINING AT WALTHAM.

Last week we gave a summary of the preparatory course of the Waltham Training School in the United States, and as some of our readers may like to know in what respects the three years' training, which follows the preparatory course, varies from that of the English training schools, we mention the more important differences. The probationers or students who enter upon the regular course at Waltham, are required during the first year to attend daily lectures or lessons five afternoons of the week. They may be at work in the wards of the hospital, or for the District Visiting Association, or in nursing the private patients of their "physician instructor." In the hospital they are under the instruction of the matron, the head nurse, and the nurse in charge of the operating room; in district nursing they are taught by the instructor of visiting nursing, and if assigned to private nursing, they are constantly visited at their cases by the instructor in that department. When they are in the Waltham Hospital they have service first as ward nurses, and then as assistant head nurses in charge of the male and female wards, and of the scarlet fever and diphtheria wards, each of which is really a separate hospital, and also as students in the operating room. Here, it may be observed, that there are no medical or surgical house officers in the Waltham Hospital. Occasionally, probationers in their second or third year have service in other hospitals. In the departments of district visiting nursing, or private nursing, the probationers or students are changed about, "not according to their special fitness, but rather according to their need of further training in these special departments." A remark in the report from which these facts are taken supplies the key to the principle on which the system of training has been founded. It is observed that "the utilitarian custom of subordinating training schools to hospitals is really a modern invention." The authorities of the Waltham School appear to be in favour of subordinating hospitals to training schools.

### NERVE-TIRED WOMEN IN CAMP.

We are asked to state that "a summer camp on very simple lines for nerve-tired women will be held during this month and September in a quiet country village near the yachting town of Burnham-on-Crouch." It will be under the general supervision of a nurse, and nurses will be welcomed. Arrangements will, it is hoped, be possible for sketching, riding parties, cycling, photographing, music, yachting, and bathing. For the information of any nurses who may desire to join the party we may add that for further particulars application should be made to Miss Goodyer, The Hut, Latchingdon, Maldon, Essex. But we must confess that a summer camp composed of nerve-tired women does not seem to us to present a fascinating picture. If nerve-tired nurses want an exhilarating change, the society of persons whose nerves are in good form would be more bracing than that of a few similarly exhausted beings.

### ROYAL BRITISH NURSES' ASSOCIATION.

At the last council meeting of the Royal British Nurses' Association Mr. E. A. Fardon resigned the position as medical honorary secretary, which he has held since 1895. A vote of thanks to him for his valuable services during that period was passed, and will be presented to him, engrossed on vellum, at the next council meeting.

### THE MEMORIAL OF A GOLDEN WEDDING.

The Victoria Nurses' Home at Ripon, now in course of erection, which is to be a memorial of the golden wedding of Lord and Lady Ripon, will have accommodation for six nurses, a matron, and an assistant matron. The building will be of brick and blue slate, and the cost will be about £1,300, inclusive of furnishing. At the recent ceremony of laying the foundation stone, Lord Ripon expressed his own gratification, and that of his wife, that the subscribers had selected a form of memorial which was calculated to confer many benefits upon their fellow citizens.

### BAZAAR AT GRIMSBY.

The Grimsby and District Nursing Institution has for some time been in rather low water financially. The bazaar which was recently held at the residence of Alderman Sutcliffe, for the purpose of rendering assistance, was both enjoyable and successful. The total amount of the receipts was £244, and it is estimated that at least £230 will be placed to the credit of the institution. This is a handsome sum, and the managers deserve praise for the manner in which they kept down the expenses and yet invested the bazaar with attractions in the shape of music and dancing.

### HOUSE-TO-HOUSE COLLECTIONS.

At the annual meeting of the Huddersfield and District Victoria Sick Poor Nurses' Association it was stated that a house-to-house collection had been started, and that nearly £50 had been obtained by this means in the Lindley district. It is undoubtedly an excellent method, and should be adopted in all cases, especially among the class for whose benefit district nursing associations are called into existence. There are thousands among working men and their wives who, though they would never send a few pence to the treasurer, would willingly give them to a district collector with a card. The Huddersfield Association, with its superintendent and five nurses, is doing admirable work. In the financial year which has just closed 13,196 visits were paid as against 12,364 the previous year, but the expenditure was £11 in excess of the income. This, however, is a small balance to put right, and if the house-to-house collections are steadily persisted in for twelve months we fully expect that the next report will show not only that the deficit has been wiped out, but also that there is a balance to the good.

### Lectures to Nurses on Medical Nursing.

BY EVELINE A. CARGILL, M.D.BRUX., L.R.C.P.&S.ED.

### LECTURE III.—DISEASES OF THE DIGESTIVE ORGANS.

(*Continued from p.* 212)

IN all cases of acute illness the nurse should pay special attention to the condition of the digestive organs as evidenced by the state of the mouth—lips, tongue, teeth. In health the tongue is moist, red, with papillæ visible but not unduly prominent. A furred tongue shows the stomach is out of order, and is common in the early stages of fever; in the later stages it becomes dry and brown. A "raw" tongue is characteristic of diabetes.

The teeth and mouth must be kept very clean. An alkaline mouth-wash is generally agreeable to the patient, such as bicarbonate of soda and water, or Vichy water, and occasionally it should be thoroughly cleaned with borax and glycerine. Lint or soft rag should be used for cleaning the mouth rather than cotton-wool. After taking acid medicine or one containing iron, the mouth should be rinsed out, as these medicines are very injurious to the teeth.

The nurse should observe if digestion appears to proceed naturally, or if there is discomfort after food, and of what kind, whether it amounts to pain; if there is retching and vomiting, and in the latter case the frequency of the act and the appearance of the vomited matter; if it consists chiefly of undigested food, curdled milk, etc., and how long after food the vomiting occurs; if any abnormal substance is mixed with it, such as blood; if the blood is bright-coloured, or dark like coffee grounds. If blood is in any quantity it should be measured. Then the odour should be noted, if acid, fœtid or fæcal; fæcal vomiting is characteristic of intestinal obstruction and means that the disease has entered on a very serious stage.

Again, the nurse should observe the frequency and characters of evacuations from the bowels. If the stool is healthy and formed, or loose or liquid; if the latter, does it contain fæcal matter, or is it a watery discharge (cholera) or slime and blood (dysentery)? Note the colour, if normal brown or pale clay-coloured (jaundice), or black (iron, bismuth), or does it contain any unusual substance, such as blood, which may be bright-coloured, and is then distinct from the fæces, or mixed with the fæces and then like tar? or is there pus, mucus, intestinal worms of any sort, gallstones. When the latter are suspected the fæces should be covered with water, and then broken up with a stick and allowed to settle. On the liquid being poured off carefully the stones will be found at the bottom of the vessel, or the contents may be passed through a sieve, the gallstones remaining behind. Lastly, is there any special odour about the stool (pea-soup stools of typhoid with characteristic odour, or the peculiar fœtor of dysentery). When anything unusual is found in the vomit or stool it should be kept, if possible, for the doctor's inspection, the vessel being covered with a towel wrung out of disinfectant solution, and then with a fitting lid of glass or crockery. By careful observation, the nurse will give the doctor very valuable help, and in some cases assist to an accurate diagnosis.

Diseases of the digestive organs, stomach, bowels and liver, are marked by disturbance of the functions of digestion as shown by discomfort or pain in stomach, abdomen or back, flatulence, nausea and vomiting, diarrhœa or constipation. Pain in the stomach is most severe in acute dyspepsia, gastric ulcer, or cancer. Severe pain is in itself serious, as it exhausts the nervous system, and must be relieved. Hot applications, poultices or fomentations are ordered for the relief of pain, and should be made as light in weight as possible and covered with a sheet of thin protective. They must be renewed at frequent intervals to keep up the sensation of heat. Hot applications must be withheld when there is any sign or risk of hæmorrhage (gastric ulcer). Vomiting is Nature's method of getting rid of what the stomach cannot digest, and is often beneficial and not harmful, but excessive vomiting must be checked or it may exhaust the patient, partly through the inability to retain food, partly through the violent muscular efforts involved. Frequent vomiting is common in intemperance and in various diseases of the brain, kidneys, uterus, etc., as well as in those of the digestive organs. Habitual vomiting in hysteria is peculiar in this, that the patient does not rapidly waste. Excessive vomiting requires great care in feeding, very small quantities should be given at a time, a teaspoonful of milk, or milk and soda, peptonised milk or whey, or sometimes only hot water, given very hot (beware of burning lips or tongue) can be retained, small pieces of ice, or effervescing drinks (champagne). When a start has once been made the quantity may be very gradually increased until the stomach becomes more tolerant, but for some time much care must be exercised to proceed very gently.

Diarrhœa from any cause requires rest, warmth, and special diet. When it is severe the patient must be kept in bed, on bland food (milk with cornflour or arrowroot) given cold; and a broad flannel bandage round the abdomen is helpful, and should be worn even when the patient is getting about again. An astringent enema is sometimes ordered for diarrhœa, it may be of starch and opium; it should be prepared from starch mixed in the usual way with boiling water, and then allowed to cool. Mix 2 oz. of this with 30 minims of tincture of opium, and administer cold.

Habitual constipation should be combated by simple methods, viz., regular habits and diet, leaving drugs to be relied on only when other means fail. Constipation is the cause of much ill-health, and is often due simply to want of care. Foremost in importance is regularity in attending to the call every day at the same hour; next comes daily exercise in the open air, and alteration in diet to suit each case. Sudden and absolute constipation is a symptom of obstruction of the bowels.

Gastric ulcer requires careful nursing. It occurs mostly in girls in a chronic form which may become acute at any moment. The serious symptoms are severe pain coming on soon after food, and vomiting of blood (hæmatemesis). The blood is generally bright-coloured and in large quantities. Hæmorrhage from the stomach is always dangerous; the patient must be kept absolutely quiet, lying down, and preferably without moving from the spot where the attack occurred. No food must be given by the mouth, and no stimulants. The patient may die from loss of blood, or from perforation, the floor of the ulcer giving way, and the contents of the stomach being poured into the abdomen. This generally fatal accident may occur at any moment and is always a possible danger. The signs are those of *collapse*; the patient experiences a sharp, sudden pain, and quickly becomes cold, clammy, and pulseless. Until the doctor can arrive the treatment is to keep the patient warm with hot blankets round each limb and hot bottles, lying quite flat, with no pillow, and with a good supply of fresh air.

In gastric ulcer nutrient enemata are useful, the object being to give the stomach complete rest, and so give the ulcer time to heal.

### The Nurses of University College Hospital.

INTERVIEW WITH THE MATRON: BY OUR OWN COMMISSIONER.

The appointment of Miss H. E. G. Hamilton to the post of matron of St. Thomas's Hospital suggested a visit to North London or University College Hospital in order that I might be able to give an account of the work which has been going on under her auspices at the institution in Gower Street. Before I left I had the opportunity of inspecting some of the fine wards of the new wing of the hospital now in course of erection, the spacious kitchens, the laundry, and the nurses' home. The home, though conveniently connected with the hospital by means of a bridge on each floor, is quite distinct from it, and the kitchen and storerooms used by the nurses are also shut off from the main building. It will be easier to form an accurate opinion of the arrangements of the latter when it is completed, but, so far as can be judged at present, the nurses will find their duties considerably lightened when the old, inconvenient wards are utterly abolished.

But it was the past, rather than the future, that formed the subject of my conversation with Miss Hamilton, who, as stated in The Hospital of last week, was appointed matron of University College Hospital in June 1899.

### The New Departure.

"You started," I said, "with a clean slate?"

"Yes; prior to my appointment the nursing, as you know, was done by All Saints' Sisterhood. The rules which are now in force were drawn up by the Hospital Committee, and are of course entirely different from those which were previously in force."

"Your next step, I conclude, was to obtain the services of a sufficient number of ward sisters?"

"We engaged thirteen who, with the night superintendent, came from other hospitals. Some of them are still with me, others have obtained appointments elsewhere, and several have gone out to South Africa. The number has not been altered."

"And the number of staff nurses and probationers?"

"We have now thirty-eight staff nurses and thirty-nine probationers. The distinction is that the former are in their third and fourth years of training, and the latter in the first and second. All the staff have, of course, been trained by me."

"Have you always had sufficient applicants?"

"There has never been any lack of applicants, but there has always been the usual difficulty in getting the right kind of nurse. Still, I have had a fair number to choose from, partly, perhaps, because of the new building.

"The greatest drawback," continued the matron, "with which I had to contend was when, after the demolition of a part of the old building, the nurses had to live in different houses in different streets. Since the home was opened a year and a half ago there has been no trouble in that respect."

### The Probationers.

"You have two classes of probationers, paying and non-paying. Are the regulations similar?"

"The most important difference is that the paying probationers are bound for three years and the non-paying for four. There is also a difference in the age of admission. Paying probationers are admitted from 23 to 35, and non-paying from 23 to 33. Then there is a difference of salary. The non-paying probationers receive £8 the first year, £12 the second, £16 the third, and fourth according to duty. The paying probationers, who are asked for a fee of £30 upon admission, have no salary the second year, but are paid £18 the third."

### The Sisters.

"I see that in the rules for the paying probationer it is stated that in the selection and appointment of sisters preference will be given, other things being equal, to such persons who have been paying probationers."

"That applies also to non-paying probationers, but so far, the system having only come into force in July 1899, there has been no opportunity of offering a probationer a sister's post. The sisters must not be under 28, nor over 38, at the time of their appointment. The salary varies from £35 to £50. The rules for sisters are much the same as at other hospitals. Each sister keeps an inventory of the beds, bedding, linen, kitchen utensils, and crockery issued to the ward; a record of patients; she has herself to take part in the nursing of the patients, also to supervise the work of the nurses. Once in every month she has to send the matron a written report on the conduct and capacity of each nurse and probationer. Among other things she has to see that there is no waste in the ward, especially in the matter of electric light and gas."

### Training and Duty.

"I suppose you have the usual lectures?"

"The training is very much on the lines of St. Thomas's. Lectures are given by members of the staff on anatomy and physiology; the ward sisters give practical instruction, and examinations are held in June. The probationers divide their time equally between the surgical and medical wards, and gain experience in the out-patients' department and the theatre. The probationers have night duty for about two months in the year, and the staff nurses three months alternately."

"How many nurses are on duty in each ward at night?"

"A staff nurse and a probationer, a night sister having charge of the whole of the wards. At present we labour under the disadvantage of having the old and new wards going at the same time. When we get the fourth block built, the nursing staff will be increased."

"To what extent?"

"We have now 185 beds, but we shall then have nearly 300, and we shall require about 20 more nurses. The wards will all be large, containing 24 beds each, instead of varying in size, as they do at present, from 6 to 16 beds."

"What time do the night nurses go on duty?"

"They breakfast at 8.30, and go on duty at 9 P.M. They come off at 8 in the morning, and have dinner at 8.30. The diet is much the same as that of the day nurses. The day nurses breakfast at 6.30, and go on duty at 7. They have two hours off duty during the day, one evening off in the week, from 6 to 10, and a day off once a month. The probationers and staff nurses are entitled to three weeks' holiday, the sisters to a month."

### The Home.

"Have you sufficient accommodation for all the nurses in the Home?"

"No, not quite. There are 84 bedrooms in the Home and

4 in the Hospital. I should explain that 20 servants sleep in the top floor of the Home.

"Where do the nurses dine?"

"In the Hospital. There will be a new dining-room in the new building. As you have seen, there is a large sitting-room for the nurses and a smaller one for the sisters in the Home, but no meals are taken there."

A PRIVATE STAFF.

"Is the formation of a private staff contemplated?"

"The plans for a private nursing home have been prepared, and the building will be proceeded with before long. One floor of it will be reserved for the use of the Hospital nurses, for whom we have no accommodation in existing circumstances.

### Presentations.

NOTTINGHAM AND NOTTS PRIVATE NURSING ASSOCIATION.—Miss Edden, the lady superintendent of the Nottingham Private Nursing Association, whose resignation is much regretted by the nurses, was presented by them on July 24th with an afternoon tea service and silver spoons as a mark of their love and esteem.

DISTRICT HOSPITAL, WEST BROMWICH.—Miss E. W. Stephens, charge nurse of the male and children's wards of the District Hospital, West Bromwich, has been presented with a handsome morocco leather writing-case from the nurses of the hospital. She is leaving to take up similar duties in a surgical home.

### English Sisters and Boer Leaders.

A CORRESPONDENT at the Sisters' Quarters of the Refugee Camps, Bloemfontein, writes us under date of June 13th: The news of peace has been a matter of the greatest joy to all of us here at the Refugee Camp. We could not at first believe that such good news could be true, but when two days later eight of the ex-commandants, who had been at the Vereeniging Conference, visited the camp, afterwards coming to the sister's quarters, and we actually saw and centre is the hospital matron, surrounded by the Boers. On her left hand, seated in a chair is General Nieuwhout; next to him, sitting, is General Brand, the General's Secretary, Mr. Van Selin squatting on the ground before him. On the left of the seated general stands the camp matron, and by her side one of the assistant camp matrons, while behind—his face partly hidden by her cap—is the camp superintendent.

spoke to these weather-beaten warriors, we began to realise that hostilities had indeed ceased. The hospital matron invited any of the ex-commandants who visited the camp on the next occasion to lunch with us, and the Boers at once accepted the invitation; so that a day or two after, eleven of the Boer ex-commandants arrived. The camp superintendent, the doctor, and almost the whole camp staff were asked to meet them, and altogether between thirty and forty of us sat down to lunch. After luncheon a photograph of us all was taken, of which I enclose a copy. In the

One of the Dutch hospital nurses can be seen at the extreme left of the photograph, a Dutch girl in the characteristic "cappie" standing near her. The other sister in a straw hat, appearing at the doorway of the dining-room, is a Dutch sister, a Hollander, who is camp matron of the small or North Camp. The whole population of the Refugee Camp does not at present number more than 6,000 persons, and as there is very little sickness we are by no means understaffed. Now that peace is proclaimed some of us expect to be recalled.

### The Quarantine Station of El Tor.

BY ONE OF THE NURSING STAFF.

The pilgrimage to Mecca has been a very large one this year, about 100,000 pilgrims visiting the sacred shrine, of these nearly 40,000 coming from the north. Now that the season is practically over, an account of the measures that have been successfully taken to fight the threatened epidemic of cholera may prove of interest. Two objects have been kept in view: one to prevent any pilgrim from returning to Egypt without passing through Tor, and the other to stamp out the epidemic by fighting it in the most efficacious manner at the quarantine station, and allowing the pilgrims to leave only when they are considered incapable of carrying infection.

### The Village of El Tor.

El Tor is situated about 120 miles S. of Suez, on the eastern coast of the Arabian Gulf. It lies in a wide plain between the sea and the cliffs of the Sinaitic range, which limits the horizon at about 13 or 14 miles inland. Steamers from Suez take from 12 to 15 hours to reach Tor, but caravans coming over the desert take five or six days to accomplish the journey. The quarantine camp stretches about two miles along the shore, and is swept constantly by strong breezes, often amounting to gales. The little village of Tor consists of a group of sand-coloured, flat-roofed houses clustering round a small Greek orthodox church, whose priest cultivates the only flower and vegetable garden in the place, and this is, fortunately, within the quarantine enclosure and so available for members of the staff. On the arrival of steamers they are visited by the quarantine doctors. All healthy pilgrims are disembarked in large boats, towed to the landing-stages, and after certain preliminaries are taken to the sections. The sick are conveyed straight to the hospital enclosure, and the dead brought ashore and buried. Then the ships and everything on board are thoroughly disinfected, and during the rest of their stay are constantly visited by the doctors.

### Disinfecting the Pilgrims.

The camp consists of bathing and disinfecting houses, sections, hospitals, offices, storerooms, dairy, tents for the president, the staff, the servants, and the soldiers. When the healthy pilgrims from a ship are disembarked they are taken first to the bathing and disinfecting houses, one of which is reserved for women, and is in charge of an Englishwoman. Their goods and clothing are put in numbered sacks, which are subjected to a heat of 120° F. for twenty minutes in the disinfecting ovens. But everything which would be spoilt by heat is soaked instead in a solution of corrosive sublimate. Meanwhile, the pilgrims are taken into spacious dressing rooms and there provided with long calico garments before passing on to the bathrooms. Here they have a choice of hot or cold douche, or hot or cold sea-water bath, for which a special soap is provided. The bath over, they go on to adjoining rooms, where their disinfected clothes and goods are handed back to them, and while they are dressing the quarantine doctor visits them, and a list of their names is taken by the passport authorities. The pilgrims are passed through in batches of 40, each batch occupying about an hour. After these preliminaries are gone through, the goods are put upon a small train kept running between the different parts of the encampment, and all the pilgrims from one ship follow to the section destined for them.

### The Sections.

A section forms a rectangle, 200 metres long by 50 metres wide, enclosed by a high fence and separated on both sides from the next section by a vacant plot of similar dimensions. The ground is laid out in four rows of 25 tents, each tent to hold six pilgrims. At the corners are the tents for the doctor, sanitary guards, and the representative of the Ministry of the Interior. Each section contains a general provision store, a restaurant, and the necessary sanitary provisions. The pilgrims are visited individually twice a day by the doctor of the section, who sends any sick to hospital. In cholera times the period of quarantine is 15 days, provided that the health of a section remains good; but if at any time a case of cholera occurs in a section the whole period of quarantine begins again. For Egyptian pilgrims the period is 18 days, as they have no long sea voyage to undergo before reaching the point of disembarkation. From the moment a section is occupied everyone inside is strictly isolated, and communication is only possible with the outer world by telephone or through the fence, a cordon of soldiers surrounding each section, and the whole camp being again encircled by a high fence strictly guarded by soldiers. The entire plain in which the camp is situated is watched by posts of Bedouin stationed on commanding points of the hills at the back, and at night watching is rendered easy by a system of bright electric lights throughout the length and breadth of the camp. There are 40 sections, of which 20 are intended to be left vacant, while the others are full, to give time for disinfection after occupation. The stores in the sections are well supplied and goods are sold at a fixed tariff, which is printed and posted up in different languages. For destitute pilgrims the Government provide two meals daily, consisting of soup, bread, and cooked lentils, rice, beans, and other vegetables.

### The Hospitals.

There are three hospitals built of masonry, two containing 30 beds each and a small bedroom for the doctor in charge, and the third containing two wards of 10 beds, one ward reserved for women and one for surgical cases. There is a well-appointed, up-to-date little theatre, and also two extra rooms which came in very useful this year for members of the staff who, unfortunately, broke done. Near the gate of the hospital enclosure is the bathing and disinfecting house, and close by is the laundry. There is also a well-fitted bacteriological laboratory. There is an enclosure fenced off and containing wooden huts with adjustable ventilating shutters, and containing two beds each. This was used for isolating cholera and other contagious cases. Yet another enclosure contains tents for housing suspected cases. As all these different enclosures became full it was necessary to erect tents until there were about 50 with two patients in each. The hospital buildings and tents are much scattered and cover a large space of ground, causing long walks in the sand to the staff going its rounds. These are the director, five or six other doctors, and a bacteriologist, trained European sisters (English, German, and Greek), and Arab nurses, both male and female.

### A Nursing Sister's Day.

To get a good idea of the work I will, in imagination, follow a sister through a day's duty. Tea is brought by her own native servant, a general factotum, at 5 A.M., and in half an hour she begins by taking the patients' temperatures. Before the night "taumurgiehs" (nurses) go off duty at 6 A.M. it is necessary to see that beds and tables are left clean; then comes the first meal of tea, coffee, milk, and bread. The doctors usually make their rounds quite early and give orders for the day. Then comes half an hour for the sisters' breakfasts, and after that the medicines must be sent for and given, milk given round, linen counted and exchanged for clean, and any treatment ordered carried out. At 12 o'clock the second meal is brought round, and consists of milk, soup, bread, cooked vegetables, and stewed meats. Next comes the sisters' own luncheon time, all meals being provided by themselves and taken in their own tents. After luncheon an hour's rest is necessary on account of the great heat. By that time it is requisite to give more milk, medicines, and treatment, and to overlook the taumurgiehs to see that everything is kept as clean as possible. At 5 the doctor make another round; at 6 comes the third meal of the same character as the mid-day one; then there are final medicines to be given, and directions for the night taumurgiehs, and very often new patients to attend to. It may be 7, but is often 8 P.M. before everything is finished, and then comes dinner time, and soon after the very welcome bed-time. The sisters try to arrange to take each other's duty in the afternoon, so that each one may get a little spare time; but on account of the work this is often not feasible, and each sister may have 40 or 50 patients under her care.

### Night Duty.

Each sister in turn does a week's night duty, and her chief work is to see that all patients get milk, and that the nurses do not sleep. It is weird work tramping about with a lantern from hospital to hospital, tent to tent, but it is a rest in one way, inasmuch as there is relief from the blazing sun and incessant winds. These two elements make nursing in tents very difficult: the sand is blown through all the crevices, the beds get covered, and the heat is often so intense that doors must be kept open. A terrific sand-storm rose one day and blew down many tents: the patients had to be carried into the hospitals as quickly as possible and laid on mattresses on the floors. Luckily, none of them was much hurt. The pilgrims are for the most part very old and worn out with travelling, and if they get ill they give up altogether. It is one of their ambitions to die on the pilgrimage, for they believe they will then go straight to Paradise. So they refuse everything, turn their faces to the wall, and beg to be allowed to die—which is discouraging for doctors and nurses. Dysentery and enteritis are the commonest forms of disease, but almost all kinds are met with. The patients who recover from their various ailments get wonderfully well in the strong, invigorating air of Tor, and the staff, in spite of real hard work throughout the pilgrim season, returns sunburnt and well to civilisation once more, most of them looking forward to renewing acquaintance with Tor next year.

### Wants and Workers.

Will anyone give or lend to a trained nurse, who is suffering from a tuberculous stiff knee-joint and quite unable to walk or do anything for a living, a self-propelling chair, to enable her to move about in the house or the garden? The case is a very deserving one, and a chair would be greatly appreciated. Address Dr. W. Duncan, Eythorne, Kent.

### To Nurses.

We invite contributions from any of our readers, and shall be glad to pay for "Notes on News from the Nursing World," or for articles describing nursing experiences, or dealing with any nursing question from an original point of view. The minimum payment for contributions is 5s., but we welcome interesting contributions of a column, or a page, in length. It may be added that notices of appointments, entertainments, presentations, and deaths are not paid for, but that we are always glad to receive them. All rejected manuscripts are returned in due course, and all payments for manuscripts used are made as early as possible after the beginning of each quarter.

### Everybody's Opinion.

**[Correspondence on all subjects is invited, but we cannot in any way be responsible for the opinions expressed by our correspondents. No communication can be entertained if the name and address of the correspondent are not given as a guarantee of good faith, but not necessarily for publication. All correspondents should write on one side of the paper only.]**

### THE RELIGIOUS QUESTION.

"Observer" writes: I have read with interest the correspondence on the subject of nurses' religion which has appeared in your columns from time to time. I know of more than one nurse, who, on being driven from her training school by the "objections" of those in command, has found a happy home, and (what all religious women prize far more highly) perfect religious freedom and every facility for the practice of her religion, under the wise and just rule of the Metropolitan Asylums Board. If all hospitals were thus governed, nurses would have very little to complain of.

### A TRUE STORY.

"E. A." writes: When nursing in a rustic village in the south of England I became attached to an old lady whom I had nursed through a serious illness. When she was better I induced the patient to attend divine service. The old lady arrived in church gaily attired in a scarlet cloak and black poke bonnet. After the service the vicar inquired how she had enjoyed it. She replied that she had been unable to hear at such a distance. The vicar then very kindly told her to sit in his seat next Sunday. Feeling sure of a good seat that day, old Mary started early for church, and as the congregation arrived they were amused to see her in her scarlet cloak in the pulpit, where she sat perfectly composed and smiling. The vicar, after prayers, ascended the pulpit stairs and at once grasped the situation. He delivered his discourse, then politely handed old Mary down the steps and requested her next Sunday to try his pew.

### THE CENTRAL MIDWIVES' BOARD.

"J. Wilson," president of the Incorporated Midwives' Institute, 12 Buckingham Street, Strand, writes: During and since the passage of the Midwives Bill a good deal has been stated both verbally and in the press to the effect that the friends of trained midwives have not sufficiently insisted that there should be at least one midwife on the Central Midwives' Board. We know of no friend of the trained midwife in this matter except the Midwives' Institute; and those who, like myself, have worked on this question for the last twelve years, can alone realise how powerful the opposition has been against the nomination of even one medical practitioner by the Midwives' Institute. Though constantly threatened by members of Parliament and by powerful medical organisations, that if we persevered in our demand for even this minimum of representation the Bill would be wrecked, we took a firm stand on the important principle that the class legislated for should have some representation, with the result that now for several years this very small claim has been conceded—and it should be added—conceded owing to the recognition of the justice of the claim, and not because at the eleventh hour the influence of important personages and parliamentary obstructive tactics were brought to bear on the question. The final effort to displace our representation came in the second reading in the House of Commons from the Royal British Nurses' Association, an entirely unexpected quarter. Those of our critics who consider that the interests of the trained midwife have not been sufficiently studied, should now use their influence to ensure that those bodies that have the power, viz., the Queen Victoria's Jubilee Institute, and the Royal British Nurses' Association should appoint fully-trained and certificated midwives as their nominees. It may be noted that there is a possibility of four midwives being nominated on the Central Board, though the fact remains, and it is due to the tactics of the opposition mentioned before, that not one of them can be nominated by the Midwives' Institute.

### BLACKBURN NURSING ASSOCIATION.

"The Hon. Secretary of the Blackburn Nursing Association" writes: I have had my attention called to your reference to the above association in The Hospital of July 26th, and am quite at a loss to know where you can have got the information you publish, as it is most incorrect. It is not, and never has been, the intention of this association to try and raise funds by a "private nursing branch." There has been a long-felt want in the town for a daily visiting nurse, at a small charge, for those in better circumstances who do not wish to come under any obligations to the charity, and it is to meet several appeals for such a nurse that the association has undertaken to try for one year (and to continue if found a success) a daily visiting nurse for those patients who can pay. The fees, however, are so small that it is not likely they will cover the cost of the nurse, and impossible that the association could ever "augment the funds" by them. The number of visits paid by the nurses from June 1st, 1901, to May 31st, 1902, was 18,632.

[The report on which our remarks were founded was taken from the *Northern Daily Telegraph*, and reference is distinctly made to "the experiment of having a nurse for paying patients." In these circumstances we considered it worth while to warn the Blackburn Association of the probable drawbacks of anything in the shape of a private nursing branch. We are glad to learn that this is not intended; but it is better for an organisation which employs Queen's Nurses not to receive fees at all and to collect small subscriptions from people who can afford to contribute than to make a departure which is capable of misconstruction.—Editor, Hospital.]

### OUTDOOR UNIFORM.

"A Queen's Nurse" writes: I have taken great interest in the letters regarding nurses wearing outdoor uniform which have appeared in the last two issues of The Hospital. For district nurses while on duty it is absolutely necessary. I quite agree that it would not be nice nor advisable to do district work in anything but uniform, and nothing could be so useful or becoming as a neat cloak and bonnet, which can so easily be taken off. I think that the writer in The Hospital of July 19th will find that as a rule nurses wear outdoor uniform from choice, as very few hospitals in these days compel their nurses to do so. If private patients who object to nurses wearing it would tell the institution from which they obtain a nurse that they objected, I am sure arrangements would be made for the nurse to wear ordinary attire. There are, it is true, some nurses who look anything but a credit to their profession in outdoor uniform; at the same time, any nurse who is devoted to her work will take sufficient pride in the mark of her calling to make it easy for an outsider to recognise her as a hospital nurse and not as a nursemaid. It is asked, why do nurses keep their outdoor uniform during their holidays? Simply because they wish to do so. The same applies in almost every case, except perhaps the district nurse. I too, have heard nurses in trams and omnibuses, in uniform, discuss in loud tones professional matters, and blush to think that some of my professional sisters have not enough respect for their work to make matters that need discussion a subject of conversation for duty time, and not by talk for off-duty hours. In the opinion of the general public a nurse is not like the average woman, and she is expected never to need recreation or amusement of any kind. They cannot realise we are but human after all. Therefore do not let us give them opportunity for comment by going to theatres, concerts, etc., in uniform. I have had varied experiences in my nursing career, have done hospital, private, and district work, and must say that on several occasions I have been glad of the protection of a cloak and bonnet. I have had the honour of wearing the Queen's uniform in the lowest parts of one of our large cities, and in a small mining district in South Wales, where most of the inhabitants had never seen a nurse in uniform before. They stared at me in astonishment, and some of them told me afterwards that they wondered who I was. One of my patients told me that he saw me the first night I came, and thought I must be some distinguished person to be dressed like that. Shortly afterwards I was stopped by a man who had occasion to be grateful to a Queen's nurse, because he thought he recognised my uniform, and he was surprised and pleased to meet a Queen's nurse in a small village in South Wales. It rests in a great measure with nurses themselves whether they get respected when they are in uniform. I have worn uniform myself for several years, and have always had proper, or even greater, respect paid me while I was wearing it than in ordinary dress. If the matrons of the institutions who wish their nurses to wear outdoor uniform would adopt a badge to be worn outside the cloak, the staff would be publicly recognised by it. It may be the badge and brassard worn by the Queen's nurses which make a Queen's nurse known by her uniform in almost every part of the United Kingdom.

### The Nurses' Bookshelf.

Guy's Hospital Nursing Guide and Register of Nurses. (Ash and Co., Limited, 42 Southwark Street, S.E. Price 1s. 6d.)

It is a distinct advantage to the nurses who are trained at Guy's Hospital that they should have a register of their own; and we have no doubt that the manual which has just been published will be welcomed by all who are, or have been, associated with the institution. The register, which is, of course, compiled alphabetically, includes the names both of nurses who hold the three years' certificate, and also of pupils who have been awarded a certificate for one year's training, the difference being clearly specified. It would appear that the subsequent career of many of the nurses who were trained at Guy's is unknown to the compilers of the register; but this, perhaps, is a deficiency which may be remedied in a future volume. The rules and regulations of Guy's Hospital Nurses' League form a portion of the manual.

The Child-Healer. By George H. R. Dabbs, M.D. With Frontispiece by Miss Lisa Stillman. (Shanklin, I.W.: Silsbury Bros. Price 6d.)

The dainty fancy and sympathy with humanity which was shown in Dr. George Dabbs's last volume, "The Dream," again appears in the little story before us. This also may be regarded as a dream story; at least it is one of those tales which assume conditions outside the knowledge of ordinary man. The proem gives the keynote of the story. A child has entered into heaven, but as he bowed before the Deity, "God saw upon his face a sadness that subdued the joy of heaven," and knew that "the child felt that if permitted to return to earth he could, with his heavenly experience, lighten the lives of children still on earth." His unspoken prayer is granted, he is permitted to return to the earth for seven days, and the tale contains the narrative of that week of divine service. The incidents are simple enough. The Child-healer restores health to a little girl, comforts his own sorrowing mother cheers his little blind sister, gives a day of happiness to slum children and the like; but the simple facts are so treated as to keep ever before the reader's mind that divine love for humanity which is not only the motive power of all our charity, but the only thing that makes it a thing which "blesseth him that gives and him that takes." We are moved not by what the child did, nor even by the magical success which attended his efforts, but by the spirit which inspired them, and gave them value not only in the eyes of those who profited by them but in those of the Divine Master who permitted them. Dr. Dabbs has the gift of true dreaming, and his dreams are always worth listening to.

### Appointments.

[No charge is made for announcements under this head, and we are always glad to receive, and publish, appointments. But it is essential that in all cases the school of training should be given.]

CHESTERFIELD AND NORTH DERBYSHIRE HOSPITAL.—Miss Florence Charlotte Scott-Smith has been appointed staff nurse. She was trained at the Jaffray Hospital, Birmingham, where she has since been assistant nurse. She has a certificate for massage.

FULHAM INFIRMARY.—Miss Hettie Louisa Philp has been appointed superintendent of night nurses. She was trained for three years at Leeds General Infirmary, and has since been Queen's nurse for 18 months, and charge nurse at the Grove Hospital, Lower Tooting.

PARISH INFIRMARY, PORTSMOUTH.—Miss Ives has been appointed charge nurse. She was trained at the Parish Infirmary, Portsmouth.

ST. ANNE'S CONVALESCENT HOME FOR CHILDREN, HERNE BAY.—Miss Elizabeth Forsyth has been appointed matron, She was trained at the Royal Hospital for Sick Children. Edinburgh, and at the Royal Infirmary, Dundee. She has since been sister at the children's ward, Mildmay Hospital, Bethnal Green, night superintendent at the Southwark Infirmary, East Dulwich, and lecturer on nursing for the School Board, London. She has also done private nursing, and for the past three years has been home sister and assistant matron at the London Temperance Hospital.

### TRAVEL NOTES AND QUERIES.

SWITZERLAND FOR THREE WEEKS (£10).—You give neither proper name or pseudonym. Kindly attend to rules. The sum is very small for three weeks, because the journey is expensive. Second return to Lausanne (nice and bracing) is £4 13s. 6d. Living there from 6 to 7 francs per day. Lucerne is not relaxing in the higher parts like the Gütch, and would be delightful; terms about the same. Interlaken would be rather cheaper; journey the same. Meringen and Grindlewald, also, would be possible, but three weeks is rather a strain on £10. Write me again as to locality preferred, and I will send you addresses.

ACCOMMODATION IN A FARM ON THE EAST COAST (Reta).—Write to the Traffic Manager at Liverpool Street and ask him to send you their tourist guide and time table. Tell him you want that which has a list of lodgings and farmhouses. You will find an endless list to choose from, sometimes with terms, usually without. We do not keep a list of lodgings, it would open too wide a field of inquiry and we have no means of verifying statements. There are some nice little places round Cromer, but Cromer is itself very expensive. Several people have spoken to me favourably of Mundesley-on-Sea, but I do not know it personally; it is fairly cheap as to lodgings, and the railway fare return third-class is 20s., return fare third to Cromer the same, and a return ticket for 15 days 15s.

### Novelties for Nurses.

BY OUR SHOPPING CORRESPONDENT.

### THE MONTHLY NURSE'S ASEPTIC OUTFIT.

(DOWN BROS., 21 ST. THOMAS'S STREET, BOROUGH, LONDON, S.E.)

AT the suggestion of Dr. George Elder, consulting surgeon to the Samaritan Hospital for Women, Nottingham, Messrs. Down Bros. have constructed a very complete monthly nurse's aseptic outfit. The object aimed at has been to provide means for carrying about in an aseptic and easily sterilisable form the various appliances required by the monthly nurse.

Often it is to be feared the scissors, the ligatures, the enema, etc., are carried from case to case jumbled together in a bag which by prolonged use becomes the opposite of aseptic; indeed it requires but little consideration to see that when the ordinary leather bag has once been fouled it is next to impossible to sterilise it. In this outfit, on the contrary, not only is there a place for everything, but the whole affair being constructed of japanned tin it can be easily sterilised at the end of each case. As is shown in the illustration it consists of a compact and portable box opening in a very ingenious way so as to render the contents of its various compartments accessible all at the same time. It contains a syphon douche with patent glass vaginal and rectal pipes, glass female catheter, clinical thermometer, pair of spring dressing forceps, pair of plain dressing scissors, pair of McBurney's indiarubber gloves (for septic and doubtful cases), two bottles in metal cases (1) for a 1 in 1,000 solution of mercuric perchloride in glycerine for lubricating the fingers, (2) sterilised silk for ligation of cord, safety pins in metal box, bottle of mercurial pellets, metal dredger for boracic powder, nail brush in metal case (a fresh nail brush should be used for each case), 12 temperature charts. The japanned tin case is fitted outside with a neat brown waterproof canvas cover.

No one can doubt the great utility of having all these things so well arranged as they are in this box, in addition to the immense advantage of being able to make all absolutely clean before going to another case Cleanliness is the keynote to success in midwifery, and the possession of sterilisable apparatus is essential. We think it would be a good plan to have a card giving definite directions as to the best method of sterilising the box and its contents enclosed in the case. The enamel looks so nice that we can well believe that many a nurse might hesitate to boil it.

### Echoes from the Outside World.

### The Coronation.

The reports of the King's progress are quite satisfactory. His health is spoken of by his physicians as "excellent," and he is able to be moved from his couch to a wheeled chair for a few hours every day. The wound is said to be healing rapidly. The Common Crier and Serjeant-at-Arms for the City of London, on Monday proclaimed August 9th as a Bank Holiday from the steps of the Royal Exchange. The King signed the necessary proclamations at a privy council held on board the *Victoria and Albert*, at Cowes, on Saturday. The regulations of the Metropolitan Police for the Coronation Day have also been published, and it appears that out of thirty-seven streets which had barriers erected for June 26th, only seven will be thus barred on August 9th. These erections are now spoken of as "gates" not "barriers." Westminster Abbey will not be opened till 7 A.M. instead of 6.30 A.M. and will also close half an hour later than it would have done on June 26th. The general public will have to be in their places about the same time as was previously announced.

### Premature Coronation Festivities.

In total ignorance of the illness of the King and the postponement of the Coronation, the residents of the colony of British Honduras proceeded with their programme of festivities. On Wednesday evening, June 25th, the Governor and Lady Wilson gave a ball at Government House, and on Thursday, June 26th, from early morning until late at night, rejoicings were kept up. There was a review of the Volunteers, who commenced to assemble at 7.30; at 10, special services were held in all the churches; and at 2 the school children marched in procession to Government House, where they were suitably entertained. In the evening the town of Belize, which was beautifully decorated with flags and arches, was illuminated. It was a great shock to the Governor and the inhabitants to learn, on Sunday, June 29th, on the arrival of the mail steamer from New Orleans, of the King's serious illness.

### "Dying for My Queen."

On Sunday afternoon, Mr. Brodrick, Secretary of State for War, unveiled a handsome mural tablet which has been placed in Holy Trinity Church, Guildford, to the memory of the officers, non-commissioned officers, and men belonging to Guildford and neighbourhood, who have fallen in the war in South Africa. In the course of his moving address, he gave a striking illustration of the gallantry of our soldiers. Weeks of misery, Mr. Brodrick states, were endured without a word of complaint; and he proceeded to say, "I was struck only a few days ago with an instance which reached me, an authentic instance, not one of those studied speeches or improved effects, but just the message of one young soldier of the Royals, who died, soon after Ladysmith, of enteric fever, and who, after he had been two or three days unconscious, at the moment of death had one of those glimpses of inspiration which we are privileged to see in some before life goes. He just turned to the nurse and sent this message, 'Tell mother not to fret, because I am dying for my Queen.'"

### The Future of South Africa.

Mr. Chamberlain made his re-appearance in the House of Commons on Tuesday for the first time after his accident, and was loudly cheered by all parties. In the debate upon the vote for the Colonial Office, the right hon. gentleman made a very important speech in which he foreshadowed the policy of the Government in South Africa. He said that a more tremendous task had never been placed on a Government than to regulate the condition of things in that country. Order had to be evolved from chaos, enmities buried, and the country restored to a condition of prosperity. Sympathy between former foes had to be created, a great portion of the Boer population repatriated, and a system of taxation, just to all, established. Mr. Chamberlain warmly eulogised the services of Lord Milner, to whom, he affirmed, the Government looked as a most effective instrument in the future. Subsequently, he declared that the Commission just appointed to revise sentences under martial law had the approval of the Cape Government and the Premier of Natal, and it was hoped that the King would exercise clemency in a large number of cases.

### The Generals in the City.

The honorary freedom and livery of the Cutlers' Company was presented on Monday to General French together with a sword of honour. The sword has an ivory handle, and on the blade are the names of the principal engagements at which the General was in command. The scabbard is tastefully ornamented with gold, and is engraved with the letters, "South Africa, 1899–1902." The Master Cutler, in presenting the sword said that it was probable no man had done so much as General French to win from the Boers respect for Britons, and that was one of the most priceless services which he had rendered to his country. General French in replying said that more than once both officers and men had been encouraged by the splendid leadership of their chiefs. He said that the great lesson to be learned from the war was the necessity of a very high order of peace training. Subsequently, Sir Ian Hamilton, in responding for the Army, said that a better man to fight under, or alongside of, than General French they could not imagine. He assured his hearers that if a bullet were to hit the wall over his head the General would only smile. On one occasion he was having breakfast with General French—rather a scarce thing sometimes—and a messenger, pale and perspiring, rode up and said that the bullets were flying thick over the encampment of one of the cavalry brigades. General French's only reply was to call out loudly for another mutton chop.

### Foreign.

The Special Commissioner of his Majesty's Government appointed to confer with the Commissioners of the Emperor of China on commercial subjects, has negotiated a new scheme by which "likin"—or, to spell it according to pronunciation, "lekin"—is to be abolished. "Likin" is an inland tax, imposed upon everything. Chinese products being sent abroad, or goods manufactured elsewhere and brought into the country, are all equally made to pay this tax, which is so burdensome that the merchants of Foochow told Lord Charles Beresford that the tea trade was rapidly diminishing in consequence, and would soon be destroyed altogether. It is computed that not more than one-fifth part of the likin actually reaches the provincial authorities. According to Sir James Lyle Mackay's scheme the whole of the charge, which is to be simply an addition to the import and export, will be collected by the Imperial Maritime Customs, which is said to be the one and only honestly administered department in China.

The sad death of a lady doctor is reported from Berlin Fraulein Doctor Else Neumann, after much perseverance, overcame the opposition of the Berlin University to the admittance of women to degrees, and was herself admitted as the first German lady doctor on February 18th, 1899. Since acquiring her academical honours she has devoted herself to experimental research in the field of electro-chemistry, and was last week found dead in her laboratory. It is presumed that she fell a victim to her studies, having either inhaled poisonous gases or drank a tumbler of water by mistake which contained some poisonous substance. There is not the slightest ground for believing that she committed suicide. Her brother is the African traveller, Herr Oscar Neumann, and her father was a well-known zoologist.

### For Reading to the Sick.

"THE PATH OF THE JUST IS AS A SHINING LIGHT."—*Prov. iv.* 18.

HENCEFORWARD, and for ever,
They live, live unto God;
He is their source, their object,
Their light, and their abode.
As sea-flowers in the ocean,
As white clouds in the air,
He forms them and expands them,
Is round them everywhere.

His joy is through them spreading;
His Will, their will sustains;
Joint heirs, in rich possession,
Of Christ's eternal gains.
With vision all unclouded,
They see him face to face,
Share in his intercessions,
And ministries of grace.

*C. M. Noel.*

In the Heavenly Country, "the Lord Himself is her everlasting light," and the light that is in Him streams forth upon the children of light in one unending day. Blessed permanence of that unending day, that undecaying light!

There is no night there, thank God! It is not advance and retrogression, but one unchecked progress; it is not the interchange of happiness and misery, but one unending song of the children of the day, revelling in the everlasting light. It is this stability of the Heavenly Land which marks its great contrast with the things of time.

It is towards such a life we are pressing—a life where humanity shall be beautified with the beauty of God; a life where humanity shall be glorified with the glory that is reflected on it from the Everlasting Light. It is a life in which the powers of humanity are perfectly developed, and thus developed are fully satisfied; a life the very instinct of which is the service of God; where temptation is unknown and weariness no more besets our path; a life of one unending day, of one unclouded happiness, of one unceasing joy. . . . Oh noble life of the justified on earth, ever progressing to the life of the glorified in Heaven! . . .

Unite your lives with Him Who is the Leader of the justified, as they travel towards the glory that shall be revealed, and you shall know how true and how blessed is the saying that "The path of the just is as the shining light, which shineth more and more unto the Perfect Day."

*Rev. G. Body.*

"From glory unto glory!" Thank God, that even here
The starry words are shining out, our Heavenward way to cheer!
That e'en among the shadows the conquering brightness glows,
As ever from the nearing Light intenser radiance flows.

*F. R. H.*

O God of Saints, to Thee we cry;
O Saviour, plead for us on high;
O Holy Ghost, our Guide and Friend,
Grant us Thy grace till life shall end;
That with all Saints our rest may be
In that bright Paradise with Thee.

*Archbishop of York.*

### Notes and Queries.

**The Editor is always willing to answer in this column, without any fee, all reasonable questions, as soon as possible.**

**But the following rules must be carefully observed:—**

**Every communication must be accompanied by the name and address of the writer.**

**2. The question must always bear upon nursing, directly or indirectly.**

**If an answer is required by letter a fee of half-a-crown must be enclosed with the note containing the inquiry, and we cannot undertake to forward letters addressed to correspondents making inquiries. It is therefore requested that our readers will not enclose either a stamp or a stamped envelope.**

*Books.*

(135) Will you kindly tell me what books would be most useful to study before entering a hospital; and also say if it is necessary to learn Latin?—*Nellie.*

Matrons of hospitals much prefer that their probationers should not have preconceived ideas on nursing before they enter their course of training. It is much better, therefore, to read books of general interest. No, Latin is not necessary.

I should be glad if you will tell me where I could get Percy Lewis's "Theory of Nursing" or any other book upon nursing second-hand.—*Monica.*

At the book stalls in the neighbourhood of the hospitals, or privately from a nurse who has finished with them.

1. Can you kindly tell me of a book which was advertised some time ago in THE HOSPITAL? It was by Watson, I think, and the price 12s. 6d. 2. I have not had my name inserted in the Nursing Directory; will you tell me if there is any charge?—*Vectis.*

1. You must mean the "Handbook for Nurses," by J. K. Watson, M.D., M.B., C.M., price 5s, from the Scientific Press. 2. There is no charge whatsoever for having the name entered in the Nursing Directory. The Editor will be glad of particulars at any time.

Kindly recommend me a book on the feeding of infants.—*Nurse C.*

"Infant Feeding by Artificial Means," by S. H. Sadler, price 5s., and "A Mother's Help and Guide to the Domestic Management of her Children," by P. Murray Braidwood, M.D., price 2s., can both be recommended.

*Medical Charities.*

(136) Could you give me the addresses of any medical charities likely to help the widow of a medical man, whose husband, in consequence of an unfortunate speculation, left her totally unprovided for? Her daughter has worked hard for some time as a masseuse, but she has been laid aside through illness, and the case is one of great hardship.—*A. M.*

Apply Hon. Secretary, the British Medical Benevolent Fund, 84 Brook Street, Grosvenor Square, W.; the *Lancet* Relief Fund, *Lancet* Office, Strand, W.C.; and of the Royal Medical Benevolent College, 37 Soho Square, W.

*Finger Nails.*

(137) Will you kindly tell me if there is anything which will cure a man biting his finger nails? He has tried aloes and it has done no good.—*E. M. I.*

His general health may require attention, as the habit of biting the finger nails may point to some nervous disorder. Better consult a medical man.

*Skin.*

(138) Will you give me the name and address of a skin specialist near Manchester? I want to know if electrolysis is to be relied upon and some other particulars.—*E. F. M.*

You will find the names and addresses of skin specialists by inquiring at the Manchester and Salford Hospital for Skin Diseases, Quay Street.

**Standard Books of Reference.**

**"The Nursing Profession: How and Where to Train." 2s. net; post free 2s. 4d.**

**"Burdett's Official Nursing Directory." 3s. net; post free, 3s. 4d.**

**"Burdett's Hospitals and Charities." 5s.**

**"The Nurses' Dictionary of Medical Terms." 2s.**

**"Burdett's Series of Nursing Text-Books." 1s. each.**

**"A Handbook for Nurses." (Illustrated). 5s.**

**"Nursing: Its Theory and Practice." New Edition. 3s. 6d.**

**"Helps in Sickness and to Health." Fifteenth Thousand. 5s.**

**"The Physiological Feeding of Infants." 1s.**

**"The Physiological Nursery Chart." 1s.; post free, 1s. 3d.**

**"Hospital Expenditure: The Commissariat." 2s. 6d.**

**All these are published by the SCIENTIFIC PRESS, Ltd., and may be obtained through any bookseller or direct from the publishers, 28 and 29 Southampton Street, London, W.C.**

## Figures and Tables

**Figure f1:**
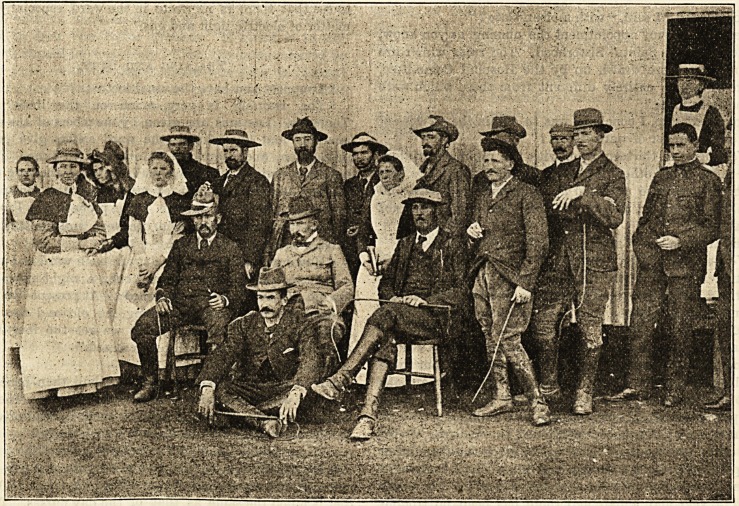


**Figure f2:**